# Should we be using nonscreening symptomatic units' mammogram machines for screening? Women's attitudes and factors likely to affect whether they attend

**DOI:** 10.1186/bcr2991

**Published:** 2011-11-04

**Authors:** EF Jones, C Zammit, G Rubin

**Affiliations:** 1 Brighton and Sussex Medical School, Brighton, UK; 2 Guy's and St Thomas's NHS Foundation Trust, London, UK; 3 Brighton and Sussex University Hospitals, Brighton, UK

## Introduction

Symptomatic mammogram machines in trusts that do not run screening services are rarely used by the National Breast Screening Service. This is a potential untapped site for screening. This study surveyed whether women attending current screening sites would go to hospital-based symptomatic units instead.

## Methods

All women who attended NHS breast screening in five different sites in South East England were surveyed over 1 day. One site was at the screening centre. The others were mobile, two being rural and two urban. Home postcodes were used to calculate how far women had travelled and the distance to the nearest symptomatic unit. This was correlated with a questionnaire about mode of transport and whether the women would be ready to attend screening at a symptomatic unit.

## Results

Women at four of the sites said they would just as likely to attend their local hospital for screening. This included one of the rural sites, where the average women would have had to travel no further to her local hospital. The fifth site was also rural but here most women would have to travel more than 15 miles to the hospital, and most had travelled less than 5 miles to the mobile unit. One woman in three would be less likely to attend screening at the local hospital than this site.

## Conclusion

Women going for screening would be happy to have this in their local hospital symptomatic unit as long as this did not entail extra travel.

